# Purification, crystallization and preliminary X-ray diffraction data of UDP-galactopyranose mutase from *Aspergillus fumigatus*

**DOI:** 10.1107/S1744309112017915

**Published:** 2012-05-23

**Authors:** George A. Penman, Deborah E. A. Lockhart, Andrew Ferenbach, Daan M. F. van Aalten

**Affiliations:** a Division of Molecular Microbiology, College of Life Sciences, University of Dundee, Dow Street, Dundee DD1 5EH, Scotland

**Keywords:** UDP-galactopyranose mutase, *Aspergillus fumigatus*

## Abstract

*Aspergillus fumigatus* UDP-galactopyranose mutase (*Af*UGM) is a potential drug target involved in the synthesis of the cell wall of this fungal pathogen. *Af*UGM was recombinantly produced in *Escherichia coli*, purified and crystallized by the sitting-drop method, producing orthorhombic crystals that diffracted to a resolution of 3.25 Å. The crystals contained four molecules per asymmetric unit and belonged to space group *P*2_1_2_1_2_1_, with unit-cell parameters *a* = 127.72, *b* = 134.30, *c* = 173.84 Å. Incorporation of selenomethionine was achieved, but the resulting crystals did not allow solution of the phase problem.

## Abstract 2

The cloning, overexpression, purification, crystallization and preliminary X-ray diffraction data are described for UDP-galactopyranose mutase, an enzyme involved in cell-wall synthesis in *A. fumigatus*.

## 1. Introduction

Medical advances have led to an expanding and diverse immunosuppressed population that is susceptible to opportunistic pathogens, including fungi. Consequently, infection in an immunocompromised host presents a spectrum of clinical, diagnostic and therapeutic challenges, often resulting in a considerable source of morbidity and mortality. Of concern is an epidemiological shift towards invasive fungal infections by *Aspergillus* species (Lass-Flörl, 2009 ▶). *A. fumigatus* (*Af*) is responsible for 90% of invasive aspergillosis (IA), in which primarily pulmonary infections can disseminate to any organ (Denning, 1998 ▶). Approximately 24–40% of at-risk patients (for example, those undergoing treatment for haematological malignancies) develop significant disease (Caira *et al.*, 2010 ▶), with mortality rates of up to 90% (Zmeili & Soubani, 2007 ▶), reflecting inherent problems in the diagnosis and treatment of IA. Voriconazole is currently regarded as a first-line therapy (Herbrecht *et al.*, 2002 ▶) for invasive disease, but there are profound drug–drug interactions, toxicity issues and initial reports of resistance (Howard *et al.*, 2009 ▶; Bueid *et al.*, 2010 ▶). Overall, this situation represents considerable medical risk, partly owing to a lack of novel antifungal drug targets in the pipeline of the pharmaceutical industry.

The fungal cell wall is a dynamic, interlaced and only partially defined polysaccharide structure that is essential for survival (Gastebois *et al.*, 2009 ▶). Like glucan and chitin, galactomannan is a major component of the cell wall in *A. fumigatus*, forming a linear core of mannan branched with short β(1–5)-linked galactofuranose (Gal*f*) side chains. Gal*f* forms the outer edge of the cell wall and is the target of a serological diagnostic test for *Aspergillus* (Stynen *et al.*, 1992 ▶). The only source of Gal*f* is by conversion from galactopyranose (Gal*p*) by the enzyme UDP-galactopyranose mutase (UGM; Trejo *et al.*, 1971 ▶; Nassau *et al.*, 1996 ▶). UGM (EC 5.4.99.9) is a flavo-containing enzyme that catalyses the isomerization of the six-membered ring (pyranose) form of galactose (Gal*p*) to the five-membered ring form (Gal*f*). Deletion of the *Af*UGM gene resulted in marked defects on solid media and a reduction in cell-wall thickness and growth rate, and attenuated virulence has been demonstrated in an animal model (Schmalhorst *et al.*, 2008 ▶). These findings were contradicted by a second report of an *Af*UGM knockout using a different strain (Lamarre *et al.*, 2009 ▶), leading to uncertainty as to the importance of UGM, and further work is required to resolve this. Crucially, UGM is absent in higher eukaryotes, making it a potential target for structure-based drug design.

The Gal*f* biosynthetic pathway has been extensively studied in prokaryotes (Richards & Lowary, 2009 ▶) and structural data have led to insights into the mechanism of prokaryotic UGM (Sanders *et al.*, 2001 ▶; Beis *et al.*, 2005 ▶; Gruber, Borrok *et al.*, 2009 ▶; Gruber, Westler *et al.*, 2009 ▶; Partha *et al.*, 2009 ▶, 2010 ▶). Although the prokaryotic and eukaryotic UGMs show less than 20% sequence conservation (Bakker *et al.*, 2005 ▶; Beverley *et al.*, 2005 ▶), the active site contains conserved residues (Oppenheimer *et al.*, 2010 ▶).

Our understanding of the structure and mechanism of *Af*UGM is very limited as there are no available crystal structures of any eukaryotic UGMs. To further characterize UGM as a potential drug target, detailed structural information on *Af*UGM is required; this communication describes the cloning, overexpression, purification, crystallization and preliminary X-ray diffraction data of *Af*UGM, including a selenomethionine (SeMet) derivative.

## 2. Materials and methods

### 2.1. Cloning and transformation

The gene coding for *Af*UGM (GenBank accession No. AJ871145.2) was obtained by PCR from a shuttle plasmid (GenScript) containing an optimized sequence using the forward primer 5′-CCTG**GGATCC**ATGACGCATCCGGACATCTC-3′ and the reverse primer 5′-TCGA**GCGGCCGC**TTACTGCGCTTTGCTTTTGC-3′. The *Bam*HI and *Not*I restriction sites are shown in bold. The PCR product was digested with *Bam*HI and *Not*I and subcloned into the pGEX6P1 plasmid, which encodes an N-terminal glutathione transferase (GST) tag and a PreScission protease cleavage site (Amersham Biosciences). Three residues (glycine, leucine and proline) remained after proteolytic cleavage of the GST tag. All plasmids were verified by sequencing (College of Life Sciences, University of Dundee).

### 2.2. Expression and purification

The pGEX6P1-*Af*UGM plasmid was transformed into *Escherichia coli* BL21 (DE3) pLysS strain and grown in Luria–Bertani (LB) medium supplemented with 50 µg ml^−1^ ampicillin. Cells were cultured at 310 K and 120 rev min^−1^ until an OD_600_ of 0.6 was reached. Expression of the protein was induced by 0.25 m*M* isopropyl β-d-1-thiogalactopyranoside (IPTG) at 291 K and 120 rev min^−1^ with an additional 48 h incubation. The cells were harvested by centrifugation at 4000 rev min^−1^ at 277 K for 30 min and the pellet was resuspended in buffer consisting of 25 m*M* Tris, 150 m*M* NaCl pH 7.5. After the addition of 0.1 mg ml^−1^ DNAse and 1 mg ml^−1^ lysozyme, the cells were lysed using a high-pressure homogenizer (Emuliflex C3, ATA Scientific) on ice. The ruptured cell debris was removed by centrifugation at 18 000 rev min^−1^ at 277 K for 1 h. The supernatant was passed through Glutathione–Sepharose 4B beads (GE Healthcare) equilibrated with lysis buffer by gravity. The GST tag was removed by overnight cleavage with PreScission protease at 10 rev min^−1^ at 277 K. The released *Af*UGM protein was further purified by size-exclusion chromatography (Superdex 75, GE Healthcare). The protein was concentrated and analysed by SDS–PAGE.

To obtain an SeMet-derivative *Af*UGM, the auxotrophic *E. coli* B893 (DE3) pLysS strain (Calbiochem) was grown in SelenoMethionine Medium Base plus Nutrient Mix (Molecular Dimensions). Following overnight culture in LB medium supplemented with 50 µg ml^−1^ ampicillin, the supernatant was removed by centrifugation. The cell pellet was resuspended in SelenoMethionine Medium containing 40 mg ml^−1^ methionine (Molecular Dimensions) and cultured (as above) for 4 h. Selenomethinone at 40 mg ml^−1^ (Molecular Dimensions) was added to the medium and after 30 min expression was induced with 1 m*M* IPTG for 4 h. Incorporation of SeMet was verified by mass spectrometry.

### 2.3. Crystallization and data collection

Commercial crystallization kits from Hampton Research and Molecular Dimensions were used for sparse-matrix screening of conditions and were followed by additive screens and further rounds of optimization to produce diffracting crystals. Crystallization was performed at 293 K using the sitting-drop vapour-diffusion method [0.5 µl protein solution (∼15 mg ml^−1^) mixed with 0.5 µl reservoir solution and equilibrated against 70 µl reservoir solution]. Initially, rhomboid crystals were observed in JCSG-plus screen (Molecular Dimensions) condition 1.7 (0.1 *M* CHES pH 9.5, 20% PEG 8000) after 24–48 h. Despite optimizing this condition, the crystals failed to diffract. Further screening trials involved varying the PEG concentration/molecular weight, buffer and introducing a salt. The resulting condition (0.1 *M* HEPES pH 7.5, 20% PEG 3350, 0.4 *M* ammonium sulfate, 4% formamide) produced two crystal forms: hexagonal rods (which failed to diffract) and plates.

*Af*UGM was cocrystallized with various substrate/inhibitor/reducing ligands, which were incubated with the protein for 10 min on ice (10 m*M* UDP-Gal*p*, 10 m*M* UDP, 5 m*M* UDP-glucose, 5–20 m*M* sodium dithionite). Ultimately, a plate-shaped *Af*UGM crystal that was cocrystallized with 5 m*M* fresh sodium dithionite and soaked in 10 mg ml^−1^ gold(I) potassium cyanide (Hampton Research) for 60 min and an SeMet-derivative crystal were employed for data collection. Each crystal was mounted on a CryoLoop (Hampton Research) and immersed into cryoprotectant (35% PEG 3350) for several seconds before being flash-cooled in liquid nitrogen. All data collections were performed at 100 K under a stream of nitrogen gas.

A data set was collected from a gold-soaked crystal to a resolution of 3.25 Å on beamline I04 at Diamond Light Source using a Q315 detector. A total of 82 images were collected with an exposure of 1.5 s and 1.1° oscillation at a crystal-to-detector distance of 421.6 mm. For SeMet-derivative *Af*UGM, beamline I24 (Diamond Light Source) was tuned to the absorption peak (12 666 eV) obtained from an X-ray fluorescence scan around the selenium edge. A data set consisting of 480 images with an exposure of 0.25 s and 0.5° oscillation at a crystal-to-detector distance of 619.4 mm was collected to a resolution of 4.0 Å. All diffraction data were integrated and scaled using *MOSFLM* (Leslie & Powell, 2007 ▶) and *SCALA* (Evans, 2006 ▶).

## 3. Results and discussion

**Figure 1:**
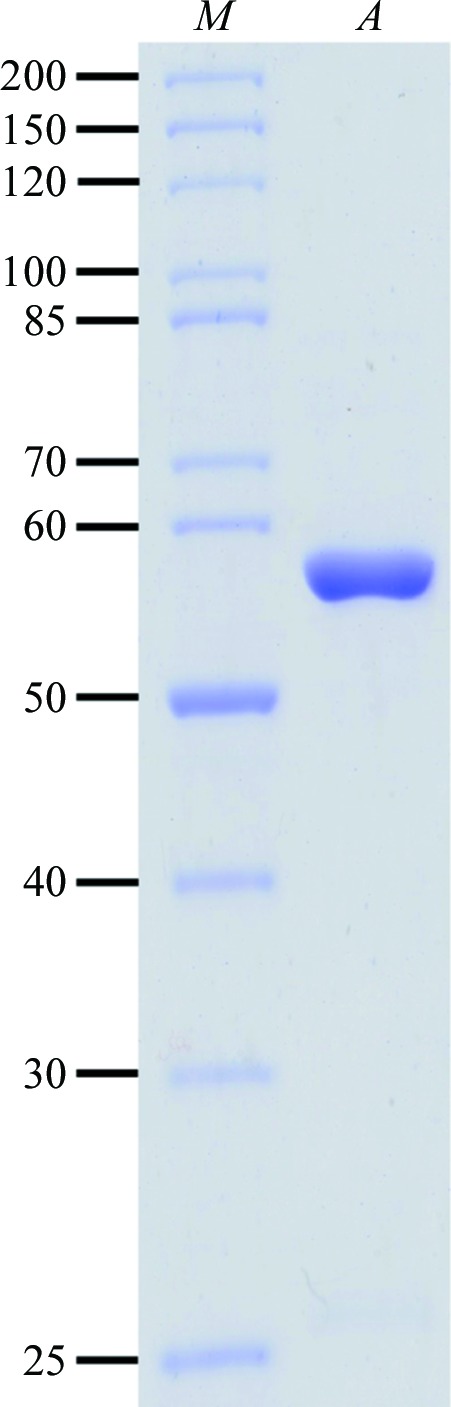
Coomassie Blue-stained 10% SDS–PAGE showing recombinant *Af*UGM purified by size-exclusion chromatography. Lane *M*, molecular-weight markers (kDa). Lane *A*, *Af*UGM.

Recombinant GST-tagged native and SeMet-labelled *Af*UGM were successfully produced in *E. coli*. After proteolytic removal of the GST tag, the proteins were purified to homogeneity by size-exclusion chromatography, eluting as a single peak; fractions that contained UGM were indicated by the characteristic yellow colour of this flavonoid-containing protein. Analysis by SDS–PAGE (Fig. 1 ▶) confirmed the presence of pure protein corresponding to the theoretical molecular mass of 57.2 kDa. Each litre of cell culture yielded approximately 4 mg pure *Af*UGM.

**Figure 2:**
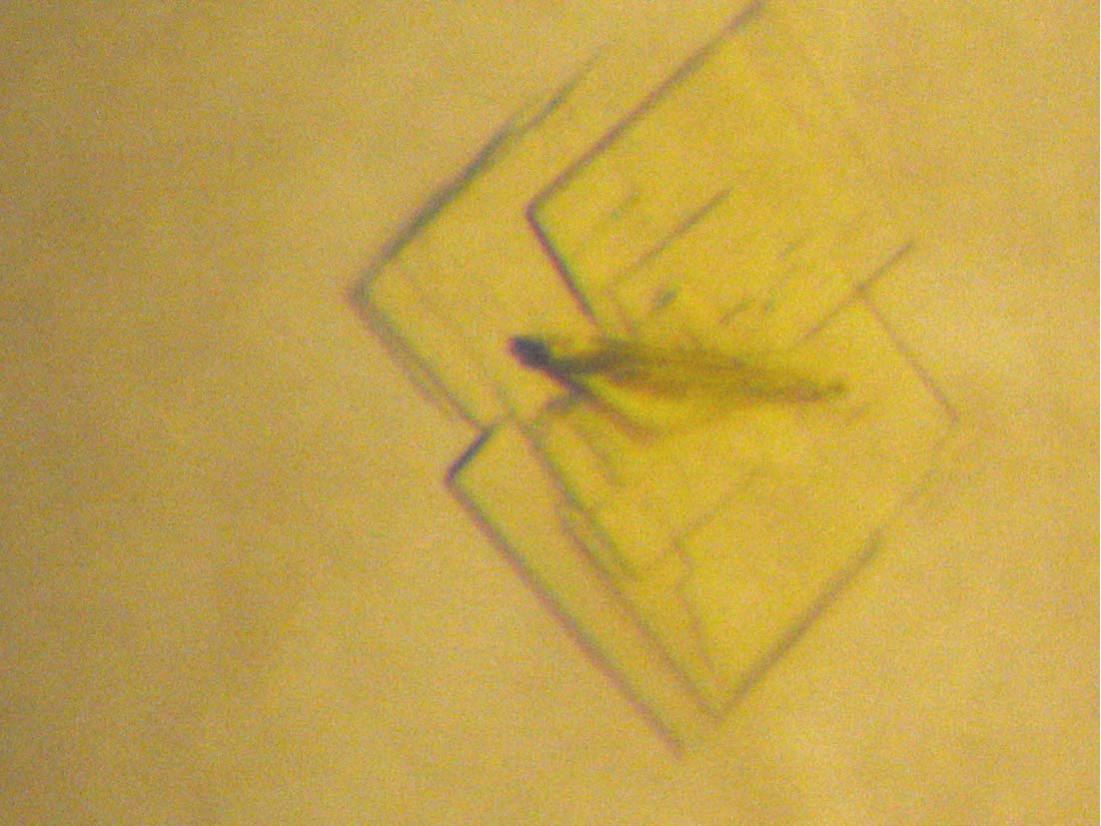
Typical plate-shaped crystal of *Af*UGM with an approximate maximum dimension of 10 µm belonging to the orthorhombic space group *P*2_1_2_1_2_1_. One of the plates was separated from the cluster before data collection.

**Figure 3:**
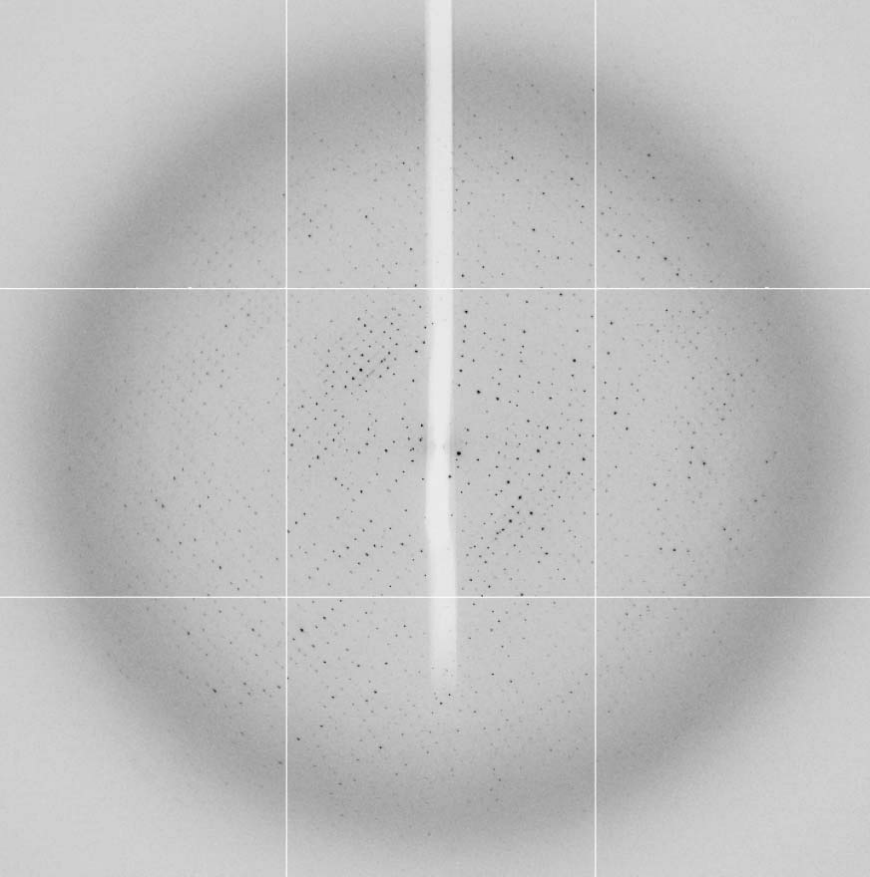
Representative X-ray diffraction image of native *Af*UGM. The crystal diffracted to 3.25 Å resolution on beamline I04 at Diamond Light Source using a Q315 detector at a wavelength of 0.96860 Å. The exposure time was 1.5 s with 1.1 ° oscillation at a crystal-to-detector distance of 421.6 mm.

Rhomboid crystals were observed within 2 d after initial sparse-matrix screening, but failed to diffract. Following further optimization, plate-shaped crystals (Fig. 2 ▶) were obtained by the sitting-drop method with a mother liquor consisting of 0.1 *M* HEPES pH 7.5, 20% PEG 3350, 0.4 *M* ammonium sulfate, 4% formamide. These were used for data collection using synchrotron radiation (Diamond Light Source). The best diffracting crystal, which diffracted to a resolution of 3.25 Å (Fig. 3 ▶), was soaked in gold potassium cyanide before data collection, but no anomalous signal corresponding to heavy-atom binding was observed; consequently, this constituted a native data set.

*Af*UGM consists of 510 amino acids, of which 15 are methionine residues. To solve the phase problem, a data set collected from a SeMet-labelled *Af*UGM crystal that diffracted to 4.0 Å resolution on beamline I24 at Diamond Light Source was processed. The data-collection and processing statistics are summarized in Table 1 ▶. Both crystals belonged to the orthorhombic space group *P*2_1_2_1_2_1_, with unit-cell parameters *a* = 127.72, *b* = 134.30, *c* = 173.84 Å for the native crystal and *a* = 127.15, *b* = 133.16, *c* = 172.40 Å for the SeMet derivative. The volume of the asymmetric unit is compatible with four molecules, as indicated by the Matthews coefficient of 3.26 Å^3^ Da^−1^ and the solvent content of 62% (Matthews, 1968 ▶). In contrast to bacterial UGMs, which are dimers, *Af*UGM has been suggested to exist as a tetramer (Oppenheimer *et al.*, 2010 ▶). Inspection of a self-rotation function did not reveal peaks that were compatible with fourfold rotational symmetry.

No significant anomalous signal was detected and attempts to locate selenium sites using a range of computer programs failed. Although the sequence homology between bacterial and eukaryotic UGM is low, conserved regions are predicted in the FAD-binding domain and active site (Oppenheimer *et al.*, 2010 ▶). To further assist with structure determination, molecular replacement was attempted using bacterial UGM structures from the Protein Data Bank (for example, *Deinococcus radiodurans* UGM; PDB entry 3he3), but this was also unsuccessful. In conclusion, this communication describes the production of recombinant crystallization-grade *Af*UGM, its crystallization and preliminary X-ray diffraction data.

## Figures and Tables

**Table 1:** Data-collection statistics for native and SeMet *Af*UGM crystals Values in parentheses are for the highest resolution shell. The statistics were as output from *SCALA* (Evans, 2006 ▶).

	Native	SeMet
Beamline	Diamond I04	Diamond I24
Temperature (K)	100	100
Wavelength (Å)	0.96860	0.97889
Space group	*P*2_1_2_1_2_1_	*P*2_1_2_1_2_1_
Unit-cell parameters (Å, °)	*a* = 127.72, *b* = 134.30, *c* = 173.84, α = β = γ = 90	*a* = 127.15, *b* = 133.46, *c* = 172.40, α = β = γ = 90
Resolution (Å)	39.80–3.25 (3.43–3.25)	39.53–4.00 (4.22–4.00)
Observed reflections	168113	191408
Unique reflections	46060	24889
Multiplicity	3.6 (3.7)	7.7 (7.6)
Completeness (%)	97.1 (98.9)	98.3 (98.1)
*R*_merge_† (%)	0.161 (0.478)	0.159 (0.384)
〈*I*/σ(*I*)〉	5.7 (2.4)	10.6 (5.0)

† *R*_merge_ = 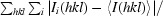 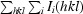, where *I_i_*(*hkl*) and 〈*I*(*hkl*)〉 are the observed intensity and the average intensity of multiple observations of symmetry-equivalent reflections, respectively.
